# Delay in seeking care for tuberculosis symptoms among adults newly diagnosed with HIV in rural Malawi

**DOI:** 10.5588/ijtld.17.0539

**Published:** 2018-03

**Authors:** L. G. Ngwira, D. W. Dowdy, M. Khundi, G. L. Barnes, A. Nkhoma, A. T. Choko, M. Murowa, R. E. Chaisson, E. L. Corbett, K. Fielding

**Affiliations:** * HIV & TB Group, Malawi-Liverpool-Wellcome Trust Clinical Research Programme, Blantyre, Malawi; † Liverpool School of Tropical Medicine, Liverpool, UK; ‡ Center for TB Research, Johns Hopkins School of Medicine, Baltimore, Maryland; § Departments of Epidemiology, Johns Hopkins Bloomberg School of Public Health Baltimore, Maryland, USA; ¶ International Health, Johns Hopkins Bloomberg School of Public Health Baltimore, Maryland, USA; # Ministry of Health, Lilongwe, Malawi; ** TB Centre, London School of Hygiene & Tropical Medicine, London, UK

**Keywords:** TB, human immunodeficiency virus, health seeking, symptom screening

## Abstract

**SETTING:**

Ten primary health clinics in rural Thyolo District, Malawi.

**OBJECTIVE:**

Tuberculosis (TB) is a common initial presentation of human immunodeficiency virus (HIV) infection. We investigated the time from TB symptom onset to HIV diagnosis to describe TB health-seeking behaviour in adults newly diagnosed with HIV.

**DESIGN:**

We asked adults (⩾18 years) about the presence and duration of TB symptoms at the time of receiving a new HIV diagnosis. Associations with delayed health seeking (defined as >30 and >90 days from the onset of TB symptoms) were evaluated using multivariable logistic regression.

**RESULTS:**

TB symptoms were reported by 416 of 1265 participants (33%), of whom 36% (150/416) had been symptomatic for >30 days before HIV testing. Most participants (260/416, 63%) were below the poverty line (US$0.41 per household member per day). Patients who first sought care from informal providers had an increased odds of delay of >30 days (adjusted odds ratio [aOR] 1.6, 95%CI 0.9–2.8) or 90 days (aOR 2.0, 95%CI 1.1–3.8).

**CONCLUSIONS:**

Delayed health seeking for TB-related symptoms was common. Poverty was ubiquitous, but had no clear relationship to diagnostic delay. HIV-positive individuals who first sought care from informal providers were more likely to experience diagnostic delays for TB symptoms.

## Abstract (FR)

**CONTEXTE:**

Dix centres de santé primaires dans le district rural de Thyolo, Malawi.

**OBJECTIF:**

La tuberculose (TB) est une présentation initiale fréquente de l'infection au virus de l'immunodéficience humaine (VIH). Nous avonsétudié le délai entre l'apparition des symptômes de TB et le diagnostic de VIH pour décrire le comportement en matière de recherche de soins de TB chez des adultes ayant récemment eu un diagnostic de VIH.

**SCHÉMA:**

Nous avons interrogé des adultes (âge ⩾18 ans) à propos de la présence et de la durée des symptômes de TB au moment où ils ont eu un diagnostic de VIH. Les associations avec le retard à la demande de soins (défini comme >30 et >90 jours après l'apparition des symptômes de TB) ont été évaluées à l'aide d'une régression logistique multivariée.

**RÉSULTATS:**

Des symptômes de TB ont été rapportés par 416 participants sur 1265 participants (33%), dont 36% (150/416) avaient eu des symptômes pendant >30 jours avant le test VIH. La majorité des participants vivait sous le seuil de pauvreté (260/416, 63%), c'est-à-dire, 0,41 $US par membre du foyer par jour. Les patients qui ont d'abord sollicité des soins auprès de prestataires informels ont eu davantage de risques de délai >30 jours (OR ajusté [ORa] 1,6 ; IC95% 0,9–2,8) ou >90 jours (ORa 2,0 ; IC95% 1,1–3,8).

**CONCLUSION:**

Le retard à la recherche de soins pour des symptômes liés à la TB a été fréquent. La pauvreté a été générale, mais n'a pas eu de rapport clair avec le retard au diagnostic. Les individus VIH positifs qui ont d'abord sollicité des prestataires de soins informels ont été plus susceptibles de connaître un retard au diagnostic des symptômes de TB.

## Abstract (SP)

**MARCO DE REFERENCIA:**

Diez consultorios de atención primaria en el distrito rural de Thyolo, en Malawi.

**OBJETIVO:**

La tuberculosis (TB) constituye un cuadro clínico inicial frecuente de la infección por el virus de la inmunodeficiencia humana (VIH). Se investigó el lapso desde el comienzo de los síntomas de TB hasta el diagnóstico de la infección por el VIH, con el fin de describir el comportamiento de búsqueda de atención de salud en los adultos con un diagnóstico reciente de infección por el VIH.

**MÉTODO:**

Se interrogaron pacientes adultos (de edad ⩾18 años) sobre la presencia de los síntomas indicativos de TB y su duración, en el momento de recibir un diagnóstico nuevo de infección por el VIH. Se evaluó la asociación entre el retraso en la búsqueda de atención de salud (definida como >30 y >90 días después del comienzo de los síntomas de TB) mediante un análisis de regresión logística multivariante.

**RESULTADOS:**

De los 1265 participantes, 416 refirieron síntomas de TB (33%), de los cuales el 36% (150/416) había estado sintomático durante >30 días antes de la prueba del VIH. La mayoría de los participantes (260/416; 63%) vivía por debajo del umbral de pobreza (0,41 USD por miembro del hogar por día). Los pacientes que acudieron en primer lugar a prestadores de atención sin titulación oficial exhibieron mayor posibilidad de un retraso superior a 30 días (OR ajustado [ORa] 1,6; IC95% 0,9–2,8) o >90 días (ORa 2,0; IC95% 1,1–3,8).

**CONCLUSIÓN:**

El retraso en la búsqueda de atención por síntomas asociados con la TB fue frecuente. La pobreza fue un factor ampliamente distribuido en los participantes, pero no exhibió una correlación clara con el retraso diagnóstico. La probabilidad de un retraso diagnóstico frente a los síntomas de TB fue mayor en las personas seropositivas frente al VIH que acudieron en primer lugar a un prestador de atención del sector informal.

HUMAN IMMUNODEFICIENCY VIRUS (HIV) and tuberculosis (TB) are the leading causes of adult deaths due to infectious agents worldwide, especially in sub-Saharan Africa.^1^ An estimated 1.2 million (12%) of the 10.4 million people who developed TB worldwide in 2015 had HIV infection.^1^ The incidence of and mortality due to TB increased steeply with the onset of the HIV epidemic and, despite recent gains, TB remains the leading cause of death among people living with HIV.^2^ TB thus still remains a major challenge.^3^

TB symptoms are commonly associated with undiagnosed HIV at all levels of the health system.^4^,^5^ Compared with TB diagnostics, HIV rapid diagnostic tests are quick, highly accurate, available in point-of-care (POC) format and more widely decentralised. As such, it is common for HIV to be diagnosed before TB investigations have started, even when the presenting complaint is consistent with active TB. Lack of rapid POC diagnostics for TB translates into a lack of rapid decision making, thereby increasing the number of visits required for TB diagnosis, loss to follow-up and patient costs. Being investigated for TB is also associated with substantial costs to patients, both direct (e.g., food and transport) and indirect (loss of wages due to time spent seeking care). Anti-tuberculosis treatment also requires multiple facility visits,^6^ and as TB is more stigmatised than HIV, it is often less widely recognised in the community.^7^ People with symptoms such as cough may therefore delay seeking care for TB, yet seek a diagnosis for HIV. Individuals with newly diagnosed HIV thus represent a group whose initial steps in the TB care pathway can be investigated.^8^

Socio-economic factors are closely associated with health-seeking behaviour,^9^,^10^ including HIV and TB (Mann G H, To what extent can the rural poor access free tuberculosis services in Malawi? Unpublished PhD thesis, University of Liverpool, UK, 2008).^11^,^12^ In settings with free health services, such as Malawi, costs can be high relative to monthly income among the poorest sectors of society.^13^ Catastrophic costs (defined as totalling >20% of the annual income) occur mostly from the onset of TB symptoms to starting treatment, a fundamental driver being the number of clinic visits.^13^ Interventions focused at reducing the time to diagnosis could reduce not only these costs, but also transmission of both TB and HIV.

Several studies have evaluated the time from the first TB symptom to TB diagnosis.^14^,^15^ Few studies, however, have looked at patient delays from the onset of symptom(s) suggestive of TB to the time of HIV diagnosis in a formal health facility. The primary aim of the present study was to investigate the factors associated with delay from the onset of TB symptoms to HIV diagnosis of >30 days among adults newly diagnosed with HIV in a rural primary health care setting in Malawi. The secondary aim was to investigate risk factors for a delay of >90 days.

## METHODS

### Study design and setting of the parent study: CHEPETSA study

The CHEPETSA study was a cluster randomised trial conducted in 12 rural primary care clinics in Thyolo District, Malawi, among adults with newly diagnosed HIV infection (CHEPETSA, clinicaltrials.gov #NCT01450085). In this parent study, clinics (clusters) were randomised to one of two TB screening algorithms: symptom screening plus sputum smear microscopy and symptom screening plus sputum testing using the Xpert^®^ MTB/RIF assay (Cepheid, Sunnyvale, CA, USA). All study participants were screened for TB symptoms (cough of any duration, fever, recent weight loss or night sweats^16^) at enrolment. If at least one TB symptom was reported, participants were asked to provide sputum for TB diagnosis with smear microscopy or Xpert, depending on the study arm. If asymptomatic and eligible, participants were initiated on isoniazid preventive therapy for 6 months. All participants were followed for 1 year after HIV diagnosis. The primary outcome of the parent trial was all-cause mortality at 1 year from enrolment.

### Study sample for this analysis

In this analysis, we used enrolment data from participants recruited into the CHEPETSA study. Trial participants were included if they were enrolled on or before 1 April 2015 from 10 clinics (five per study arm) and reported one or more of the four TB symptoms listed above at enrolment. Baseline evaluation used standardised questionnaires to elicit demographics (age and sex), time of onset and duration of TB symptoms, asset ownership, smoking status and transit time to clinic. (See Appendix Table A for the variables used in asset ownership).^*^

### Statistical methods

We defined delay a priori as >30 days from the onset of TB symptoms to the time of HIV diagnosis. By design, HIV diagnosis occurred at the same time as enrolment into the CHEPETSA study. Using data collected by self-reporting on asset ownership, recent purchase of sugar, education level of the household head, household cooking over firewood, acreage cultivated, household size, and whether maize and/or tobacco was grown in the household, we created a ‘wealth score’ variable for household wealth that was measured using a proxy means test developed for rural populations from the 1998 Malawi Integrated Household Survey (IHS) (see Appendix). This method estimates household consumption (measured as the wealth score) using proxy measures.^17^ The wealth score was then coded as a binary variable using a predefined cut-off point of 10.47 Malawian kwacha (valued in 1998 currency, equal to US$0.41) per person per day.^17^ Participants with an estimated household consumption below this cut-off were classified as severely poor. Age was grouped into three categories: <30 years, 30–40 years and >40 years. The initial site of care seeking was dichotomised as clinic/hospital (formal services) vs. other (traditional healer/pharmacist/none). In this rural setting, pharmacists are generally not formally trained and do not dispense prescription drugs, but rather sell non-prescription drugs as well as other grocery items.

We used logistic regression to explore the factors associated with delay, including age group, sex, education, smoking status, employment, time taken to reach the clinic from home, marital status, mode of transport to the clinic, site of first attempted treatment seeking, time taken from home to the clinic and then back home, history of previous anti-tuberculosis treatment, household size, self-reported general health and wealth. We hypothesised that age group, wealth and sex would affect health-seeking behaviour,^8^,^18^ and therefore included these variables a priori in all adjusted analyses. In addition, other factors from the univariable analysis with *P* < 0.2 were included in the multivariable analysis and retained if, after adjustment, the *P* value remained <0.2. Analysis was also repeated using a secondary outcome of delay of >90 days. All analyses were performed using Stata 13 (Stata Corp, College Station, TX, USA).

### Ethical considerations

The parent trial was approved by the Malawi College of Medicine Research, Blantyre, Malawi, the Ethics Committee of the London School of Hygiene & Tropical Medicine, London, UK, and Johns Hopkins Medicine Institutional Review Board, Baltimore, MD, USA. All study participants provided written consent before enrolment.

## RESULTS

Overall, 1577 participants from 10 clinics were enrolled into the parent trial on or before 1 April 2015 (Figure). Of these, 312 were excluded from further analysis as not having complete data on socioeconomic status collected. During screening, 458 (36%) of the remaining 1307 eligible participants reported one or more TB symptoms at enrolment. Of these, a further 42 participants were excluded due to missing data on delay and/or components of the wealth score, leaving 416 participants for the analysis of delay in seeking care.

**Figure:**
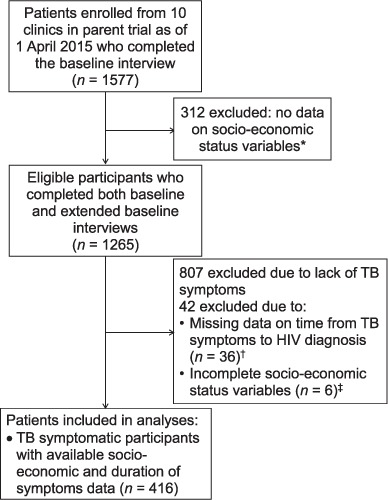
Study profile. * All socio-economic data missing (collection of these data started 3 months into the study using the extended baseline interview). ^†^ Time from the onset of TB diagnosis to HIV diagnosis missing. ^‡^ 14 socio-economic status variables were used to recreate a wealth variable using a proxy means test, and six participants had data missing on at least one of these 14 variables. TB = tuberculosis; HIV = human immunodeficiency virus.

The baseline characteristics of the 416 participants are given in Table 1. Overall, 52% of the participants were male; 60% were aged <40 years. Most participants were severely poor based on the 1998 poverty line (*n* = 260, 63%), had low levels of education (none/primary: *n* = 329, 81%) and had travelled >1 h from home to get to the clinic (*n* = 294, 71%).

**Table 1:**
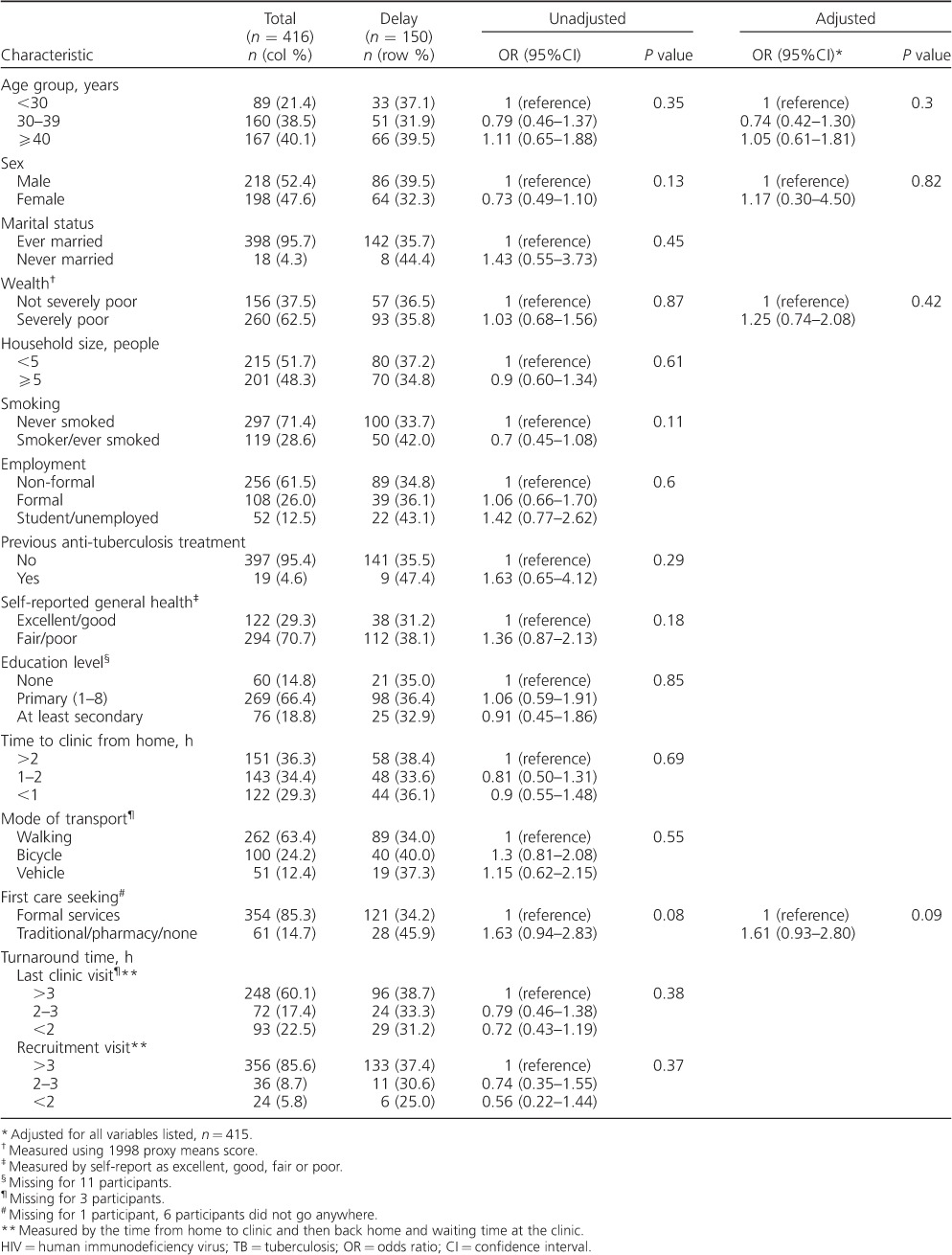
Characteristics of HIV-positive adults attending primary care clinics, and association with the delay from TB symptom onset to HIV diagnosis of >30 days (n = 416)

Of the 416 participants with TB symptoms, 150 (36%) reported a delay of >30 days from the onset of these symptoms to HIV diagnosis. Seventy-eight (19%) participants reported a delay of >90 days.

In multivariable analysis, patients who first sought care from informal (traditional healers and pharmacists) or no other services, as opposed to formal services (clinics or hospitals), had increased odds of delay; this finding was not statistically significant (adjusted odds ratio [aOR] 1.61, 95% confidence interval [CI] 0.9–2.8, *P* = 0.09; Table 2). When delay was defined as >90 days from symptom onset to HIV diagnosis, patients who first sought care from settings other than formal services (clinics or hospitals) had increased odds of delay (aOR 2.0, 95%CI 1.1–3.8, *P* = 0.03; Table 2). Patients from households with ⩾5 members (compared with those with <5 members) had lower odds of delay (aOR 0.7, 95%CI 0.4–1.2, *P* = 0.16; Table 2), although this finding was not significant. Delay—whether measured as 30 or 90 days—did not vary significantly according to age group, sex, smoking status, time taken to reach the clinic from home, wealth, mode of transport or having previous anti-tuberculosis treatment (Table 2).

**Table 2:**
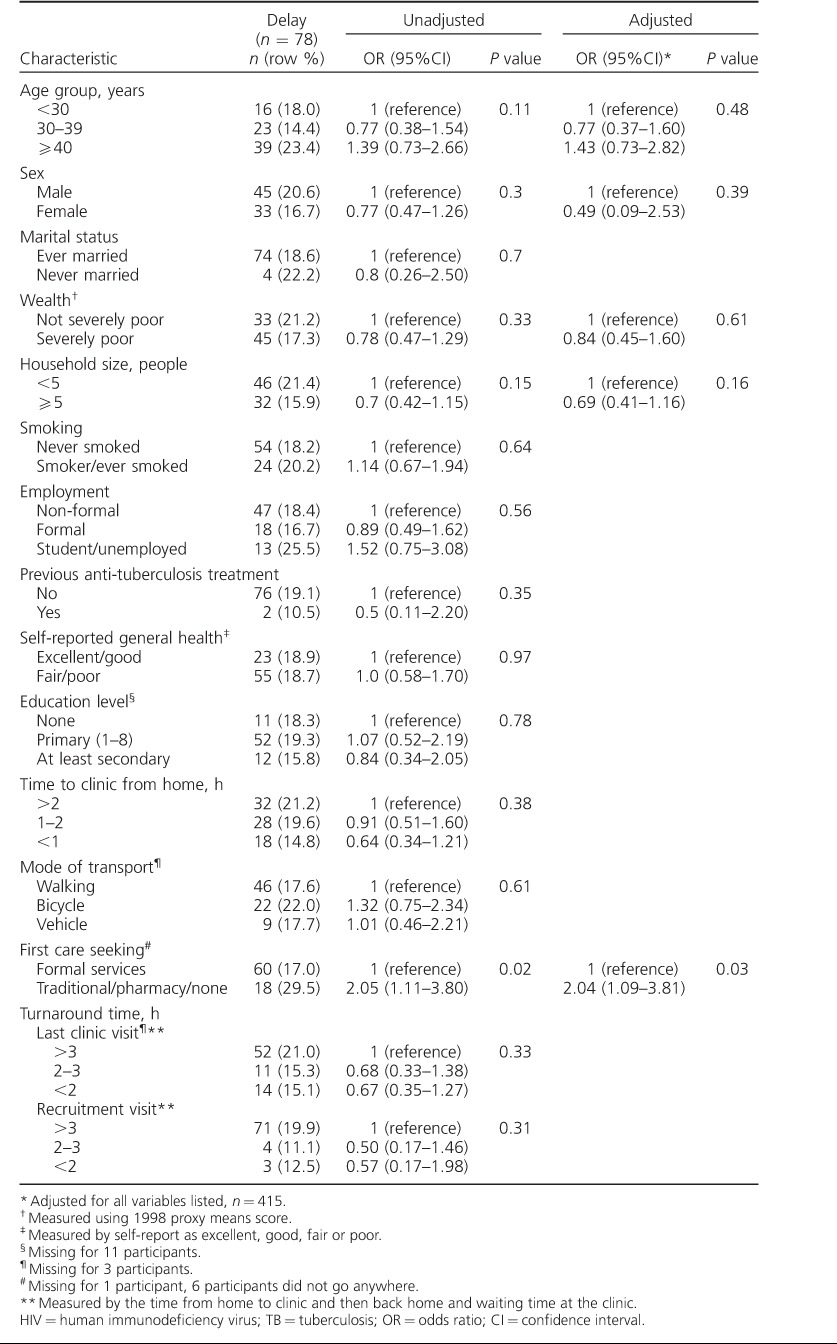
Characteristics of HIV-positive adults attending primary care clinics, and association with the delay from TB symptom onset to HIV diagnosis of >90 days (n = 416)

## DISCUSSION

This analysis of 416 rural Malawian adults newly diagnosed with HIV and reporting TB symptoms suggests that patients who first seek care from traditional healers and pharmacists may have increased odds of a prolonged delay in diagnosis. We also highlight the high prevalence of both extreme poverty (63%) and delay in diagnosis (36% with >30 days delay) in this rural population. We report the duration of TB symptoms before HIV diagnosis, and provide support for the measurement of TB symptoms at HIV diagnosis as a potentially useful approach for assessing delayed care seeking for HIV-TB.

Despite careful consideration of multiple risk factors, we found no significant associations with delayed care seeking, which was defined using our a priori cut-off of >30 days between the self-reported onset of TB symptoms and date of HIV diagnosis. When considering prolonged delays (>90 days), seeking assistance from a traditional healer or pharmacist was associated with a doubling of the odds of diagnostic delay. While only 15% of patients first sought a diagnosis from traditional healers and pharmacists (as opposed to visiting clinics or hospitals), these individuals accounted for nearly half of all patients who experienced prolonged delays. We did not find evidence of an association between first seeking treatment from traditional healers/pharmacists and any other risk factors in this cross-sectional study.

Our findings are consistent with work in Malawi by Brouwer et al., who found that 37% of TB patients visited a traditional healer before seeking formal medical care, that these patients spent an average of 4 weeks with traditional healers and that none of the traditional healers referred patients to the formal health care system.^19^ These findings suggest that individuals seeking care from traditional healers and pharmacists may be an important group for targeted case-finding interventions, and that further engagement of such informal providers may be required to reduce diagnostic delays in the rural sub-Saharan African setting. Future studies are needed to confirm this finding and also to explore the potential impact of private-sector care on population-level transmission of TB in Malawi and other similar settings.

We did not detect an association between extreme poverty and delayed care seeking for TB symptoms in this population. In most societies, the greatest burden of TB (and increasingly HIV) is experienced by the poorest populations.^20^,^21^ HIV in itself is also a powerful risk factor for progression from tuberculous infection to TB disease, and is a driver of poverty.^22^ However, despite the very high levels of poverty in our study (with 63% of the study population classified as extremely poor based on a proxy means test benchmarked to the 1998 poverty line), we found no evidence of a difference in diagnostic delays between severely poor and non-severely poor participants. In practice, the majority of participants in this study classified as not severely poor would still be classified as extremely poor by most international standards. Thus we may have failed to observe an association between wealth and diagnostic delay, in part due to the relatively ubiquitous poverty in our study setting.^23^

Different approaches have been recommended for integrating HIV and TB services so as to provide universal access even to the poorest in all societies.^24^ Systematic screening of people living with HIV and prompt treatment are the principal tools for reducing transmission and controlling the spread of TB disease.^25^ However, despite these longstanding recommendations, the integration of HIV and TB services has been slow,^26^ and many studies have indicated that much still needs to be done.^27^,^28^ The novel approach discussed here—monitoring and reporting of duration of TB symptoms before HIV diagnosis—may provide a useful and readily obtainable metric that could be used to investigate both TB and HIV programme performance relating to a prompt offer of HIV testing to all patients reporting TB symptoms. This metric can also provide an indicator of health care-seeking behaviour among people with symptoms suggestive of TB, who may be more likely to present for an HIV diagnosis than for evaluation of their TB symptoms.

The present study had five important limitations. The first was our inability to explicitly measure integration of HIV and TB services and potential recall bias in participants' self-reported time from onset of TB symptoms to HIV diagnosis. Second, data on the total number of visits made to the formal health sector before HIV diagnosis were not collected. Third, our epidemiological setting of rural Malawi—while important—is not likely to be generalisable to urban settings or to those outside of sub-Saharan Africa. Fourth, our proxy means test of household wealth benchmarked against a poverty line (i.e., an absolute measure of poverty) may not accurately reflect poverty as actually experienced in this population. Not only was this measure designed to reflect a specific context and time (i.e., Malawi in 1998), but it may be sensitive to participants' conceptualisation of household size or structure. Future studies might consider evaluating relative measures of poverty (in which wealth is compared against that of other members in the same society) to provide a different perspective. Finally, we did not include some variables (for example, seasonality) that may have important effects on the delay in seeking care.

In conclusion, we found that about two thirds of this rural Malawian population newly diagnosed with HIV and reporting TB symptoms met the criteria for extreme poverty, and one third reported delays of >30 days from symptom onset to the time of HIV diagnosis. Seeking care with informal providers was associated with an extreme delay in care seeking. These data highlight the challenges faced in diagnosing TB among people living with HIV in this setting, provide a metric (duration of TB symptoms at the time of HIV diagnosis) that can be used to evaluate programme performance, and underscore the importance of engaging informal providers if global targets for HIV and TB control are to be met in rural sub-Saharan Africa.

## Figures and Tables

**Table A:**
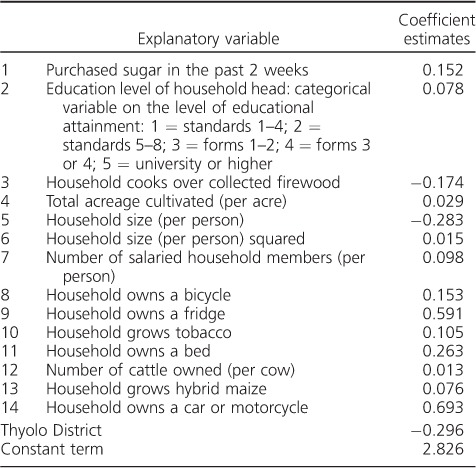
Rural Malawi proxy means test model using 1998 model by Payongayon et al.^17^

## Footnotes

^*^ The appendix is available in the online version of this article, at http://www.ingentaconnect.com/content/iuatld/ijtld/2018/00000022/00000003/art00009

## APPENDIX

The proxy means score was estimated for each participant based on the linear regression model, as follows:

where β_0_, β_1_, β_2_,..., β_k_ (*k* = 14) are coefficients provided for in the Malawi Integrated Household Survey 1997–1998 proxy means test model. The coefficient estimates and description of each explanatory variable for the rural proxy means test model are shown in Table A. In this Table, unless stated otherwise, variables are coded as binary variables, where 0 denotes a ‘no’ response and 1 a ‘yes’ response.

From this regression model a logarithm (proxy means test score) was estimated for each participant and converted back to the original scale using the anti-logarithm (exponential) function to give a predicted proxy means test score for each study participant. The predicted wealth scores (on the original scale) were grouped into a binary variable, poor/non-poor, based on a predefined cut-off point using the 1998 proxy means test model score. The cut-off point was based on the weighted poverty line for Malawi as a whole (as of March 1998), which was at Malawian kwacha 10.47 per person per day, or US$0.41.^17^ All participants with a predicted wealth score of ⩽10.47 were classified as poor and those with a score of >10.47 as non-poor.

## References

[1] World Health Organization. Global tuberculosis report, 2016. WHO/HTM/TB/2016.13 Geneva, Switzerland: WHO, 2016.

[2] World Health Organization. WHO policy on collaborative TB/HIV activities: guidelines for national programmes and other stakeholders. WHO/HTM/TB/2012.1. WHO/HIV/2012.1 Geneva, Switzerland: WHO, 2015. 23586124

[3] Pai M, Schito M. Tuberculosis diagnostics in 2015: landscape, priorities, needs, and prospects. J Infect Dis 2015; 211 Suppl 2: S21– S28. 25765103 10.1093/infdis/jiu803 PMC4366576

[4] Getahun H, Kittikraisak W, Heilig C M, Development of a standardized screening rule for tuberculosis in people living with HIV in resource-constrained settings: individual participant data meta-analysis of observational studies. PLOS Med 2011; 8: e1000391. 21267059 10.1371/journal.pmed.1000391 PMC3022524

[5] Nliwasa M, Corbett E, MacPherson P, Wright A, Horton K. The prevalence of HIV and risk of early mortality in adults with suspected tuberculosis in low- and middle-income countries: a systematic literature review. PROSPERO 2015: CRD42015021944.

[6] Barter D M, Agboola S O, Murray M B, Bärnighausen T. Tuberculosis and poverty: the contribution of patient costs in sub-Saharan Africa—a systematic review. BMC Public Health 2012; 12: 980. 23150901 10.1186/1471-2458-12-980 PMC3570447

[7] Wynne A, Richter S, Jhangri G S, Alibhai A, Rubaale T, Kipp W. Tuberculosis and human immunodeficiency virus: exploring stigma in a community in western Uganda. AIDS Care 2014; 26: 940– 946. 24521055 10.1080/09540121.2014.882488

[8] Chikovore J, Hart G, Kumwenda M, Chipungu G, Desmond N, Corbett L. Control, struggle, and emergent masculinities: a qualitative study of men's care-seeking determinants for chronic cough and tuberculosis symptoms in Blantyre, Malawi. BMC Public Health 2014; 14: 1053. 25301572 10.1186/1471-2458-14-1053 PMC4200169

[9] Topp S M, Li M S, Chipukuma J M, Does provider-initiated counselling and testing (PITC) strengthen early diagnosis and treatment initiation? Results from an analysis of an urban cohort of HIV-positive patients in Lusaka, Zambia. Int AIDS Soc 2012; 15: 17352. 10.7448/IAS.15.2.17352 PMC3494161 23010377

[10] Wingfield T, Boccia D, Tovar M, Defining catastrophic costs and comparing their importance for adverse tuberculosis outcome with multi-drug resistance: a prospective cohort study, Peru. PLOS Med 2014; 11: e1001675. 25025331 10.1371/journal.pmed.1001675 PMC4098993

[11] Kemp J R, Mann G, Simwaka B N, Salaniponi F M L, Squire S B. Can Malawi's poor afford free tuberculosis services? Patient and household costs associated with a tuberculosis diagnosis in Lilongwe. Bull World Health Organ 2007; 85: 580– 585. 17768515 10.2471/BLT.06.033167 PMC2636388

[12] Odone A, Crampin AC, Mwinuka V, Association between socioeconomic position and tuberculosis in a large population-based study in rural Malawi. PLOS ONE 2013; 8: e77740. 24204945 10.1371/journal.pone.0077740 PMC3804525

[13] Foster N, Vassall A, Cleary S, Cunnama L, Churchyard G, Sinanovic E. The economic burden of TB diagnosis and treatment in South Africa. Soc Sci Med 2015; 130: 42– 50. 25681713 10.1016/j.socscimed.2015.01.046

[14] Storla D G, Yimer S, Bjune G A. A systematic review of delay in the diagnosis and treatment of tuberculosis. BMC Public Health 2008; 8: 15. 18194573 10.1186/1471-2458-8-15 PMC2265684

[15] Sreeramareddy C T, Panduru K V, Menten J, Van den Ende J. Time delays in diagnosis of pulmonary tuberculosis: a systematic review of literature. BMC Infect Dis 2009; 9: 91. 19519917 10.1186/1471-2334-9-91 PMC2702369

[16] World Health Organization. Systematic screening for active tuberculosis: principles and recommendations. WHO/HTM/TB/2013.04 Geneva, Switzerland: WHO, 2013. 25996015

[17] Payongayon E, Benson T, Ahmed A, Simple household poverty assessment models for Malawi: proxy means test from the 1997–98 Malawi Integrated Household Survey. Lilongwe, Malawi: National Statistical Office, Government of Malawi, 2006.

[18] Ukwaja K N, Alobu I, Nweke C O, Onyenwe E C. Healthcare-seeking behavior, treatment delays and its determinants among pulmonary tuberculosis patients in rural Nigeria: a cross-sectional study. BMC Health Serv Res 2013; 13: 25. 23327613 10.1186/1472-6963-13-25 PMC3560225

[19] Brouwer J A, Boeree M J, Kager P, Varkevisser C, Harries A D. Traditional healers and pulmonary tuberculosis in Malawi. Int J Tuberc Lung Dis 1998; 2: 231– 234. 9526196

[20] Lönnroth K, Jaramillo E, Williams B G, Dye C, Raviglione M. Drivers of tuberculosis epidemics: the role of risk factors and social determinants. Soc Sci Med 2009; 68: 2240– 2246. 19394122 10.1016/j.socscimed.2009.03.041

[21] Lönnroth K, Glaziou P, Weil D, Floyd K, Uplekar M, Raviglione M. Beyond UHC: monitoring health and social protection coverage in the context of tuberculosis care and prevention. Plos Med 2014; 11: e1001693. 25243782 10.1371/journal.pmed.1001693 PMC4171373

[22] Getahun H, Matteelli A, Chaisson R E, Raviglione M. Latent Mycobacterium tuberculosis infection. N Engl J Med 2015; 372: 2127– 2135. 26017823 10.1056/NEJMra1405427

[23] Rose G. Sick individuals and sick populations. Int J Epidemiol 1985; 14: 32– 38. 3872850 10.1093/ije/14.1.32

[24] Suthar A B, Ford N, Bachanas P J, Towards universal voluntary HIV testing and counselling: a systematic review and meta-analysis of community-based approaches. PLOS Med 2013; 10: e1001496. 23966838 10.1371/journal.pmed.1001496 PMC3742447

[25] Kranzer K, Afnan-Holmes H, Tomlin K, The benefits to communities and individuals of screening for active tuberculosis disease: a systematic review. Int J Tuberc Lung Dis 2013; 17: 432– 446. 23485377 10.5588/ijtld.12.0743

[26] Getahun H, Gunneberg C, Granich R, Nunn P. HIV infection-associated tuberculosis: the epidemiology and the response. Clin Infect Dis 2010; 50 Suppl 3: S201– S207. 20397949 10.1086/651492

[27] Gupta S, Granich R, Date A, Review of policy and status of implementation of collaborative HIV-TB activities in 23 high-burden countries. Int J Tuberc Lung Dis 2014; 18: 1149– 1158. 25216827 10.5588/ijtld.13.0889

[28] Raviglione M, Marais B, Floyd K, Scaling up interventions to achieve global tuberculosis control: progress and new developments. Lancet 2012; 379: 1902– 1913. 22608339 10.1016/S0140-6736(12)60727-2

